# Four New Monoterpenoid Glycosides from the Flower Buds of *Magnolia biondii*

**DOI:** 10.3390/molecules21060728

**Published:** 2016-06-03

**Authors:** Wei-Sheng Feng, Yu-Huan He, Xiao-Ke Zheng, Jian-Chao Wang, Yan-Gang Cao, Yan-Li Zhang, Kai Song

**Affiliations:** 1 Collaborative Innovation Center for Respiratory Disease Diagnosis and Treatment & Chinese Medicine Development of Henan Province, Henan University of Chinese Medicine, Zhengzhou 450046, China; 13071020601@163.com (Y.-H.H.); zhengxk.2006@163.com (X.-K.Z.); 15238397097@163.com (J.-C.W.); caoyangang1987@126.com (Y.-G.C.); zyl2013hnzy@163.com (Y.-L.Z.); songky5901@163.com (K.S.); 2 School of Pharmacy, Henan University of Chinese Medicine, Zhengzhou 450046, China

**Keywords:** *Magnoliaceae*, flower buds of *Magnolia biondii*, monoterpenoid glycosides, biosynthetic route

## Abstract

Four new monoterpenoid glycosides **1**–**4**, named magnoliaterpenoid A–D, were isolated from a 50% aqueous acetone extract of flower buds of *Magnolia biondii*, along with one known compound, (1′*R*,3′*S*,5′*R*,8′*S*,2*Z*,4*E*)-dihydrophaseic acid 3-*O*-β-d-glucopyranoside (**5**). Their structures and relative configuration were identified by extensive spectroscopic analysis (IR, UV, MS, 1D and 2D NMR). The aglycones of these four new compounds possess seven-membered rings systems, which are very rare. A plausible biosynthetic route for the four new compounds was proposed via the biogenetic isoprene rule. Compounds **1**, **2**, **3**, and **4** showed no antimicrobial activity at the concentration range of 1.95–250 µg/mL.

## 1. Introduction

**Figure 1:**
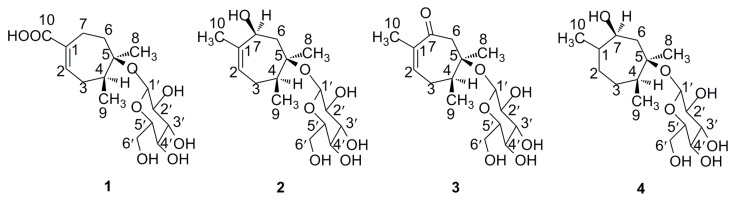
Structures of compounds **1**–**4**.

The genus *Magnolia* (family Magnoliaceae) includes about 90 species worldwide, mainly distributed in tropical and temperate regions of Asia and North America to South America. In China, there are about 30 species, mostly distributed in the southern and northern provinces of China [1]. *Flos Magnoliae*, the flower buds of *M. biondii, M. denudata or M. sprengeri,* which is unique to China and mostly distributed in the Henan, Hubei, Zhejiang, Anhui, and Shanxi provinces, has been used as a traditional Chinese medicine for more than 1500 years to treat nasal congestion, headaches, sinusitis, and allergic rhinitis [2,3,4]. Its volatile oil has also been processed into all kinds of cosmetics which are widely used in China [4]. It has been reported to possess extensive anti-allergy [5,6], anti-inflammatory [7], anti-angiogenic [8], anti-platelet aggregation [9,10], antimicrobial [11] and antioxidant activities [12,13]. Several types of compounds have been isolated from the plant, including lignans [9], neolignans [9], sesquiterpenoids [14], alkaloids [15,16], and flavonoids [15,16]. In order to further investigate the active components of the herb, a systematic phytochemical study was carried out on the 50% aqueous acetone extract of flower buds of *Magnolia biondii.* As a result, four new monoterpenoid glycosides**1**–**4**, named magnoliaterpenoid A–D (Figure 1), were isolated and structurally identified, along with a known sesquiterpenoid glycoside, (1′*R*,3′*S*,5′*R*,8′*S*,2*Z*,4*E*)-dihydrophaseic acid 3-*O-*β-d-glucopyranoside (**5**). Herein, the isolation and structural elucidation of compounds **1**–**4** are reported, as well as their antimicrobial activity.

## 2. Results and Discussion

### 2.1. Structure Elucidation of Compounds ***1**–**4***

Compound **1**, obtained as a colorless gum, was determined to have the molecular formula C_16_H_26_O_8_Cl by HRESIMS (*m/z* 381.1301 [M + Cl]^−^, calcd. 381.1310),with four degrees of unsaturation. Its UV spectrum showed an absorption maximum at 217 nm, indicating the presence of an α,β-unsaturated carbonyl moiety. The IR spectrum supported the presence of hydroxyl (3367 cm^−1^), carbonyl (1682 cm^−1^) and double bond (1652 cm^−1^) groups. The ^1^H-NMR spectroscopic data of **1** (Table 1) displayed the existence of two methyl singlets [δ_H_ 1.25 (3H, s, H-8), 1.23 (3H, br. s, H-9)] and one olefinic proton [δ_H_ 6.98 (1H, t, *J* = 2.2, 4.9Hz, H-2)] (See the Supplementary Materials). Figures S1–S36 showing the ^1^H-NMR, ^13^C-NMR , DEPT, HSQC, HMBC, ^1^H-^1^H COSY, NOESY, HR-ESI-MS, UV and IR spectra of compounds **1**–**4** can be found in the Supplementary Materials.

**Figure 2:**
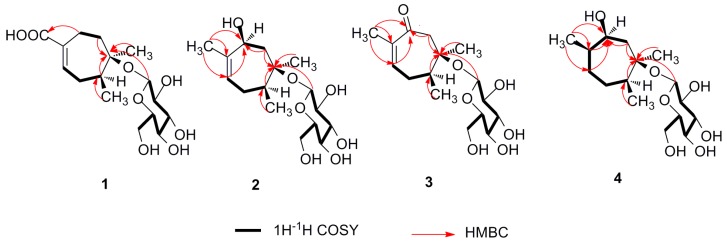
Key COSY and HMBC correlations of compounds **1**–**4**.

**Figure 3:**
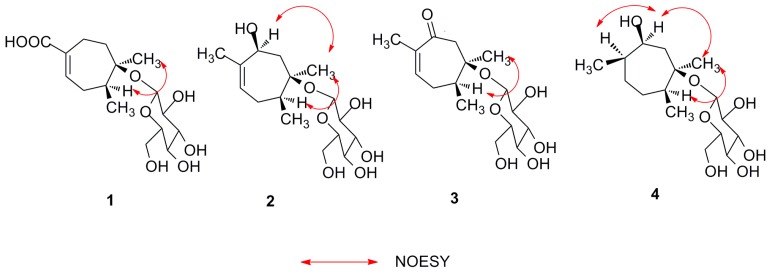
Key NOESY correlations of compounds **1**–**4**.

Closer examination of the ^1^H/^13^C/^1^H-^1^H COSY/HSQC/HMBC NMR data (Table 1) obtained for **1** revealed the presence of a glucose moiety [δ 4.46 (H-1′), 98.5 (C-1′); 3.11 (H-2′), 75.3 (C-2′); 3.21 (H-3′), 77.5 (C-3′); 3.26 (H-4′), 71.8 (C-4′); 3.35 (H-5′), 78.3 (C-5′); 3.80 (H-6′a), 3.63 (H-6′b), 62.9 (C-6′)], along with 10 carbon resonances, including two methyl [δ_C_ 24.8 (C-8), 23.3 (C-9)], three methylene [δ_C_ 28.6 (C-3), 24.5 (C-6), 26.3 (C-7)], two methine [δ_C_ 141.3 (C-2), 44.5 (C-4)], one carbonyl [δ_C_ 170.9 (C-10)], one oxygenated quaternary carbon [δ_C_ 80.5 (C-5)] and one olefinic quaternary carbon [δ_C_ 131.5 (C-1)]. Apart from one double bond, one carbonyl and one glucose moiety, the remaining unsaturation of **1** required it must contain a ring. Inspection of the ^1^H- and ^13^C-NMR spectra of **1** indicated a monoterpenoid glycoside with a structure similar to that of paeoveitol B [17]. The 7.8 Hz coupling constant for the anomeric proton H-1′ of the glucose at δ_H_ 4.46 confirmed its axial orientation. Acid hydrolysis and GC-MS analysis of the corresponding thiazolidine derivative substantiated the sugar unit as being β-d-glucose. In the ^1^H-^1^H COSY spectrum (Figure 2), the correlations of H-2/H-3/H-4 and H-6/H-7 displayed the key spin systems. In the HMBC spectrum (Figure 2), the correlation from H-8 to C-5 indicated that the 8-CH_3_ was located to C-5; the correlation from H-9 to C-4 indicated that the 9-CH_3_ was connected to C-4; the correlation from H-1′ to C-5 indicated that the β-D-glucose was linked to C-5; the correlations from H-2 and H-7 to C-10 indicated that the carbonyl (C-10) was linked to C-1; the oxygenated quaternary carbon (C-5) was located between C-4 and C-6 from the HMBC correlations from H-4 and H-6 to C-5. The relative configuration of compound **1** was established by the NOESY experiment (Figure 3) in which the correlation of H-4 and H-8 was observed. Thus, the structure of compound **1** was determined to be as shown in Figure 1, and it was assigned the trivial name magnoliaterpenoid A.

Compound **2** was isolated as a colorless gum and had a molecular formula C_16_H_28_O_7_Cl by HRESIMS (*m/z* 367.1519 [M + Cl]^−^, calcd. 367.1518), indicating three degrees of unsaturation. The IR spectrum showed the presence of hydroxyl (3345 cm^−1^) and double bond (1385 cm^−1^) groups. The ^1^H-NMR spectroscopic data of **2** (Table 1) revealed the existence of three methyl singlets [δ_H_ 1.21 (3H, s, H-8), 1.21 (3H, br. s, H-9), 1.70 (3H, s, H-10)] and one olefinic proton [δ_H_ 5.44 (1H, d, *J* = 4.2 Hz, H-2)] (See the Supplementary Materials). Comparison of its ^13^C-NMR and DEPT data with those of compound **1** indicated that the two compounds possessed a similar skeleton. One of the differences between these two compounds was that the methylene signal at C-7 (δ_C_ 26.3) in **1** was replaced by an oxygenated methine at C-7 (δ_C_ 71.9) in **2**, which was established by the HMBC correlations (Figure 2) from H-2, H-6 and H-10 to C-7, along with the ^1^H-^1^H COSY correlations (Figure 2) of H-6/H-7. The other main difference was that the carbonyl at C-10 (δ_C_ 170.9) in **1** was replaced by a methyl at C-10 (δ_C_ 19.2) in **2**, which was determined on the basis of the HMBC correlations (Figure 2) from H-10 to C-1, C-2 and C-7. The relative configuration of compound **2** was deduced from the NOESY experiment (Figure 3) in which the correlationof H-4/H-8 and H-7/H-8 was detected. Similarly, the sugar unit of compound **2** was confirmed as β-d-glucose using the same method as for compound **1**. Therefore, the structure of **2** was assigned as shown in Figure 1, and it was given the trivial name magnoliaterpenoid B.

Compound **3** was isolated as a colorless gum and had a molecular formula C_16_H_26_O_7_Cl by HRESIMS (*m/z* 365.1357 [M + Cl]^−^, calcd. 365.1361), with four degrees of unsaturation. Its UV spectrum showed an absorption maximumat 240 nm, indicating the presence of an α,β-unsaturated carbonyl moiety. The IR spectrum supported the presence of hydroxyl (3364 cm^−1^), carbonyl (1655 cm^−1^) and double bond (1368 cm^−1^) groups. The ^1^H-NMR spectroscopic data of **3** (Table 1) revealed the existence of three methyl singlets [δ_H_ 1.28 (3H, s, H-8), 1.24 (3H, br. s, H-9), 1.72 (3H, s, H-10)] and one olefinic proton [δ_H_ 6.87 (1H, d, *J* = 5.4 Hz, H-2)] (See the Supplementary Materials). The ^13^C-NMR and DEPT spectroscopic data of **3** (Table 1) were highly similar to those of **2**. The only difference between compound **2** and **3** was that the oxygenated methine at C-7 (δ_C_ 71.9) in **2** was replaced by a carbonyl at C-7 (δ_C_ 203.5) in **3**, which was established by the HMBC correlations (Figure 2) from H-2, H-6 and H-10 to C-7. The relative configuration of compound **3** was assigned by the NOESY experiment (Figure 3) in which the correlation of H-4 and H-8 was observed. Similarly, the sugar unit of compound **3** was also confirmed as β-d-glucose using the same method as for compound **1**. Therefore, the structure of **3** was established as shown in Figure 1, and the compound was trivially named magnoliaterpenoid C.

Compound **4** was isolated as a colorless gum. Its molecular formula was established as C_16_H_30_O_7_Na by HRESIMS (*m/z* 357.1878 [M + Na]^+^, calcd. 357.1883), with two degrees of unsaturation. The IR spectrum displayed the existence of hydroxyl (3362cm^−1^) groups. The ^1^H-NMR spectroscopic data of **4** (Table 1) supported the existence of three methyl singlets [δ_H_ 1.23 (3H, s, H-8), 1.14 (3H, br. s, H-9), 0.91 (3H, d, *J* = 6.2 Hz, H-10)] (See the Supplementary Materials). Critical analysis of ^13^C-NMR and DEPT spectroscopic data of **4** (Table 1) demonstrated that its structure was closely related to that of compound **2**, except that the double bond at C-1 (δ_C_ 138.2) and C-2 (δ_C_ 124.6) in **2** was reduced to a methane at C-1 (δ_C_ 37.9) and a methylene at C-2 (δ_C_ 29.6) respectively in **4**, which was established by the HMBC correlations (Figure 2) from H-10 to C-1 and C-2, along with the ^1^H-^1^H COSY correlations (Figure 2) of H-1 and H-2. The relative configuration of compound **4**was similar to compound **2**, which was deduced from the NOESY experiment (Figure 3) in which the correlation of H-4/H-8, H-7/H-8 and H-1/H-7 was detected. Similarly, the sugar unit of compound **4** was also confirmed as β-d-glucose using the same method as for compound **1**. Thus, the structure of **4** was established as shown in Figure 1, and the compound was given the trivial name magnoliaterpenoid D.

The identity of compound **5** was determined by spectroscopic analysis and comparison with literature data [18].

### 2.2. Plausible Biogenetic Pathway

**Scheme 1:**
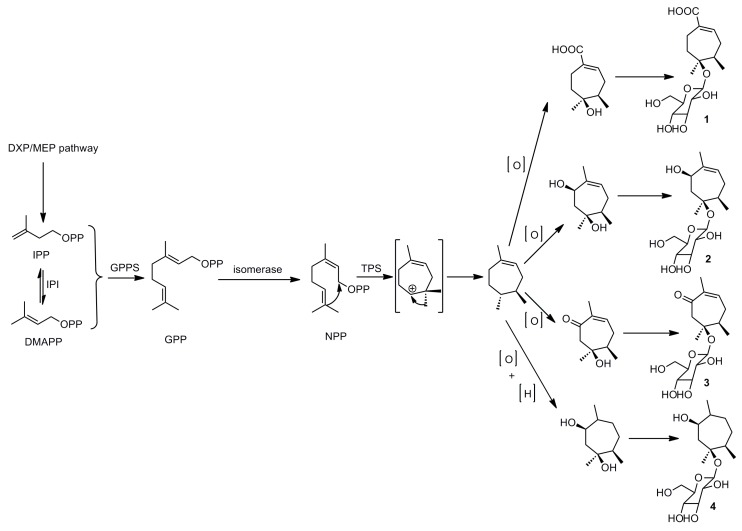
Plausible biogenetic route of **1**–**4**.

Since the aglycones of these four new compounds contained 10 carbon atoms and belonged to the monoterpenoid class of compounds, we propose a plausible biosynthetic route via the biogenetic isoprene rule, as shown in Scheme 1.

### 2.3. Antimicrobial Activity

The four new compounds were tested for their antimicrobial activity against Gram-positive and negative bacteria and fungi at the concentration range of 1.95–250 μg/mL. None of them exhibited antimicrobial activity at the tested concentrations.

## 3. Experimental Section

### 3.1. General Procedures

Optical rotations were measured on an AP-IV polarimeter (Rudolph, NJ, USA). UV spectra were measured with a Thermo EVO 300 spectrometer (Thermo Fisher Scientific, Madison, WI, USA). IR spectra were recorded on a Thermo Nicolet IS 10 spectrometer (Thermo Fisher Scientific). NMR spectra were scanned on an Avance III spectrometer (500 MHz for ^1^H-NMR and 125 MHz for ^13^C-NMR, Bruker, Zug, Switzerland). HR-ESI-MS was recorded on a Bruker MicroTOF-Q II spectrometer (Bruker Daltonics, Bremen, Germany). GC analysis was carried out on a Shimadzu GC-MS-QP2010E instrument (Shimadzu Corporation, Tokyo, Japan) using a RXI-5 ms capillary column (30 m × 0.25 mm × 0.25 µm, Restek, Bellefonte, PA, USA). Preparative HPLC was performed on a LC-3000 instrument (Chuangxintongheng, Beijing, China) equipped with a UV-3000 detector using YMC HPLC columns (5 µm, 10.0 × 250 mm and 5 µm, 20.0 × 250 mm), with a flow rate of 2.0 mL/min and 8.0 mL/min, respectively. Column chromatography was performed using DiaionHP-20 (Mitsubishi Chemical Corporation, Tokyo, Japan), ODS-B (50 µm, Daiso Co., Ltd., Tokyo, Japan), MCI GEL CHP20p (75–150 µm, Mitsubishi Chemical Corporation), and Toyopearl HW-40 (TOSOH Corporation, Tokyo, Japan). Thin-layer chromatography was carried out on self-made silica gel G (Qingdao Marine Chemical Industry, Qingdao, China) plates. The chemical reagents were supplied by Beijing Chemical Plant (Beijing, China) and Tianjin No. 3 Reagent Plant (Tianjin, China).

### 3.2. Plant Material

The flower buds of *Magnolia biondii* were collected from Nanzhao, Henan Province, China, and identified by Prof. Cheng-Ming Dong of the Henan University of Traditional Chinese Medicine, China. A voucher specimen (No. 20140609) has been deposited in Department of Natural Medicinal Chemistry, School of Pharmacy, Henan University of Chinese Medicine.

### 3.3. Extraction and Isolation

The air-dried flower buds of *Magnolia biondii* (5.0 kg) were extracted with aqueous acetone (50% *v/v*, 3 × 20 L) at room temperature. The combined solutions were evaporated under vacuum to give a crude extract (463 g). The crude extract was suspended in H_2_O (2 L) and then successively extracted with petroleum ether, EtOAc and *n*-BuOH (2 L × 5), respectively. The *n*-BuOH fraction (60.0 g) was subjected to Diaion HP-20 column chromatography (10.0 × 60.0 cm) and eluted with EtOH–H_2_O (0:100, 20:80, 40:60, 80:20, 95:5, *v/v*, each 20 L) to afford five fractions (Fr. I–Fr. V). Fr. II (14.0 g) was then subjected to ODS column chromatography (4.0 × 30.0 cm) and eluted successively with a MeOH–H_2_O (0:100, 5:95, 10:90, 15:85, 20:80, 25:75, 35:65,45:55, 100:0, *v/v*, each 1.5 L) gradient to give nine fractions (Fr. 1–Fr. 9). Fr. 6 (1.3 g) was further separated by Toyopearl HW-40 column chromatography (2.0 × 50.0 cm) with MeOH–H_2_O (25:75) as the eluent to obtain eight subfractions (Fr. 6A–Fr. 6H). Fr. 6B (50.0 mg) was further purified by preparative HPLC on a YMC HPLC column (5 µm, 20.0 × 250 mm, flow rate 8 mL/min) with MeOH–H_2_O (25:75) to afford compound **1** (4.6 mg, *t*_R_ = 77.5 min). Fr. 6C (110.0 mg) was further isolated by preparative HPLC on a YMC HPLC column (5 µm, 20.0 × 250 mm, flow rate 8 mL/min) with MeOH–H_2_O (30:70) to afford compound **2** (36.0 mg, *t*_R_ = 59.5 min). Fr. 6E (120.0 mg) was also further purified by preparative HPLC on a YMC HPLC column (5 µm, 20.0 × 250 mm, flow rate 8 mL/min) with MeOH–H_2_O (35:65) to afford compound **3** (50.0 mg, *t*_R_ = 26.0 min) and compound **4** (2.0 mg, *t*_R_ = 15.0 min). Compound **5** (12.0 mg, *t*_R_ = 60.0 min) was obtained from Fr. 6G by preparative HPLC on a YMC HPLC column (5 µm, 20.0 × 250 mm, flow rate 8 mL/min) with MeOH–H_2_O (15:85).

### 3.4. Compound characterization 

*Magnoliaterpenoid A* (**1**): colorless gum; ${\left[\alpha \right]}_{D}^{20}$ –28.8 (MeOH, 0.10); UV λ_max_ nm (log ε): 217 (0.8); IR (iTR): 3367, 2943, 2879, 1682, 1652, 1424, 1203, 1140 cm^−1^; ^1^H- and ^13^C-NMR, see Table 1; HRESIMS: *m/z* = 381.1301 [M + Cl]^−^, (C_16_H_26_O_8_Cl, calcd.381.1310).

*Magnoliaterpenoid B* (**2**): colorless gum; ${\left[\alpha \right]}_{D}^{20}$ –13.4 (MeOH,0.72); UV λ_max_ nm (log ε): 203 (0.5); IR (iTR): 3345, 2969, 2917, 2884, 1385,1367, 1073, 918, 860cm^−1^; ^1^H- and ^13^C-NMR, see Table 1; HRESIMS: *m/z* = 367.1519 [M + Cl]^−^, (C_16_H_28_O_7_Cl, calcd.367.1518).

*Magnoliaterpenoid C* (**3**): colorless gum; ${\left[\alpha \right]}_{D}^{20}$ –15.5 (MeOH, 1.0); UV λ_max_ nm (log ε): 240 (0.8); IR (iTR): 3364, 2973, 2923, 2886, 1655, 1368, 1073, 904 cm^−1^; ^1^H- and ^13^C-NMR, see Table 1; HRESIMS: *m/z* = 365.1357 [M + Cl]^−^, (C_16_H_26_O_7_Cl, calcd. 365.1361).

*Magnoliaterpenoid D* (**4**): colorless gum; ${\left[\alpha \right]}_{D}^{20}$ –6.6 (MeOH, 0.03); UV λ_max_ nm (log ε): 202 (0.6); IR (iTR): 3362, 2926, 2871, 1100, 995 cm^−1^; ^1^H- and ^13^C-NMR, see Table 1; HRESIMS: *m/z* = 357.1878 [M + Na]^+^, (C_16_H_30_O_7_Na, calcd. 357.1883).

### 3.5. Acid Hydrolysis and Sugar Analysis

Compounds **1**–**4** (1.0 mg, respectively) were refluxed with 8% HCl (2 mL) for 3 h. After the reaction mixture was extracted with EtOAc (2 mL × 3), the aqueous layer was dried under vacuum. Then the residue was dissolved in pyridine (0.3 mL) containing l-cysteine methyl ester hydrochloride (1.5 mg) and heated at 60 °C for 1 h. A 0.3 mL solution of phenyl isothiocyanate (1.5 mg) in pyridine was added to the mixture, which was heated at 60 °C for 1 h. The reaction mixture was directly analyzed by gas chromatography (GC). The d-configuration of glucose was confirmed by comparing the retention time with a standard sample [*t*_R_ (min): d-glucose (8.5)].

### 3.6. Antimicrobial Assay

The antimicrobial activity of compounds **1**–**4** was evaluated against Gram-positive bacteria (*Staphylococcus aureus* ATCC25923), Gram-negative bacteria (*Escherichia coli* ATCC35150 and *Proteus vulgaris* ATCC33420) and fungi (*Aspergillusniger* ATCC6257 and *Candida albicans* ATCC90029) by a microdilution titre technique [19]. Kanamycin and fluconazole was used as positive controls. All tests were performed in triplicate.

## 4. Conclusions

In conclusion, although these four new compounds did not show antimicrobial activity at the tested concentration range of 1.95–250 µg/mL, the aglycones of these four new compounds possess seven-membered rings system which are very rare and a plausible biogenetic pathway of **1**–**4** was proposed via the biogenetic isoprene rule. This kind of skeleton was also isolated from the *Magnoliaceae* for the first time.

## Figures and Tables

**Table 1:** ^1^H- and ^13^C-NMR data for compounds **1**–**4** (500/125 MHz, in CD_3_OD).

Position	1		2		3		4	
	δ_H_	δ_C_	δ_H_	δ_C_	δ_H_	δ_C_	δ_H_	δ_C_
1		131.5 (s)		138.2 (s)		135.8 (s)	1.40 (1H, m)	37.9 (d)
2	6.98 (1H, t, 2.2, 4.9)	141.3 (d)	5.44 (1H, d, 4.2)	124.6 (d)	6.87 (1H, d, 5.4)	148.3 (d)	1.40 (2H, m)	29.6 (t)
3	2.02 (1H, m)	28.6 (t)	1.84 (1H, m)	28.3 (t)	2.28 (1H, m)	28.6 (t)	1.01 (1H, m)	28.3 (t)
	2.41 (1H, m)		2.05 (1H, m)		2.48 (1H, m)		1.76 (1H, m)	
4	1.72 (1H, m)	44.5 (d)	1.76 (1H, m)	44.6 (d)	2.16 (1H, m)	46.7 (d)	1.93 (1H, m)	41.3 (d)
5		80.5 (s)		80.3 (s)		79.3 (s)		81.1 (s)
6	2.15 (2H, m)	24.5 (t)	1.21 (1H, m)	35.1 (t)	2.28 (1H, m)	40.4 (t)	1.23 (1H, m)	35.1 (t)
			2.27 (1H, dd, 5.7, 11.6)		2.66 (1H, m)		2.17 (1H, m)	
7	2.45 (2H, m)	26.3 (t)	4.08 (1H, br. s)	71.9 (d)		203.5 (s)	3.84 (1H, m)	71.9 (d)
8	1.25 (3H, s)	24.8 (q)	1.21 (3H, s)	24.1 (q)	1.28 (3H, s)	24.9 (q)	1.23 (3H, s)	24.1 (q)
9	1.23 (3H, s)	23.3 (q)	1.21 (3H, s)	23.6 (q)	1.24 (3H, s)	23.5 (q)	1.14 (3H, s)	23.6 (q)
10		170.9 (s)	1.70 (3H, s)	19.2 (q)	1.72 (3H, s)	15.6 (q)	0.91 (3H, d, 6.2)	18.8 (q)
1′	4.46 (1H, d, 7.8)	98.5 (d)	4.45 (1H, d, 7.8)	98.4 (d)	4.45 (1H, d, 7.8)	98.5 (d)	4.45 (1H, d, 7.7)	98.6 (d)
2′	3.11 (1H, m)	75.3 (d)	3.12 (1H, m)	75.2 (d)	3.12 (1H, m)	75.2 (d)	3.10 (1H, m)	75.4 (d)
3′	3.21 (1H, m)	77.5 (d)	3.20 (1H, m)	77.5 (d)	3.20 (1H, m)	77.5 (d)	3.23 (1H, m)	77.8 (d)
4′	3.26 (1H, m)	71.8 (d)	3.23 (1H, m)	71.7 (d)	3.26 (1H, m)	71.6 (d)	3.16 (1H, m)	72.2 (d)
5′	3.35 (1H, m)	78.3 (d)	3.34 (1H, m)	78.3 (d)	3.33 (1H, m)	78.2 (d)	3.33 (1H, m)	78.2 (d)
6′	3.63 (1H, m)	62.9 (t)	3.62 (1H, dd, 5.6, 11.9)	62.8 (t)	3.61 (1H, m)	62.8 (t)	3.56 (1H, m)	63.2 (t)
	3.80 (1H, m)		3.78 (1H, dd, 2.1, 11.9)		3.78 (1H, m)		3.81 (1H, m)	

## Supplementary Materials

Supplementary materials can be accessed at: http://www.mdpi.com/1420-3049/21/6/728/s1.
