# Supplementary material for: Four New Monoterpenoid Glycosides from the Flower Buds of Magnolia biondii
Source: Molecules. 2016 Jun 3;21(6):728. doi: 10.3390/molecules21060728 (PMC6274139; doi:10.3390/molecules21060728)
Supplement: Supplementary file 1 [file molecules-21-00728-s001.pdf]

Supplementary Materials: Four New Monoterpenoid Glycosides from the Flower Buds of *Magnolia biondii*

Wei-Sheng Feng, Yu-Huan He, Xiao-Ke Zheng, Jian-Chao Wang, Yan-Gang Cao, Yan-Li Zhang and Kai Song

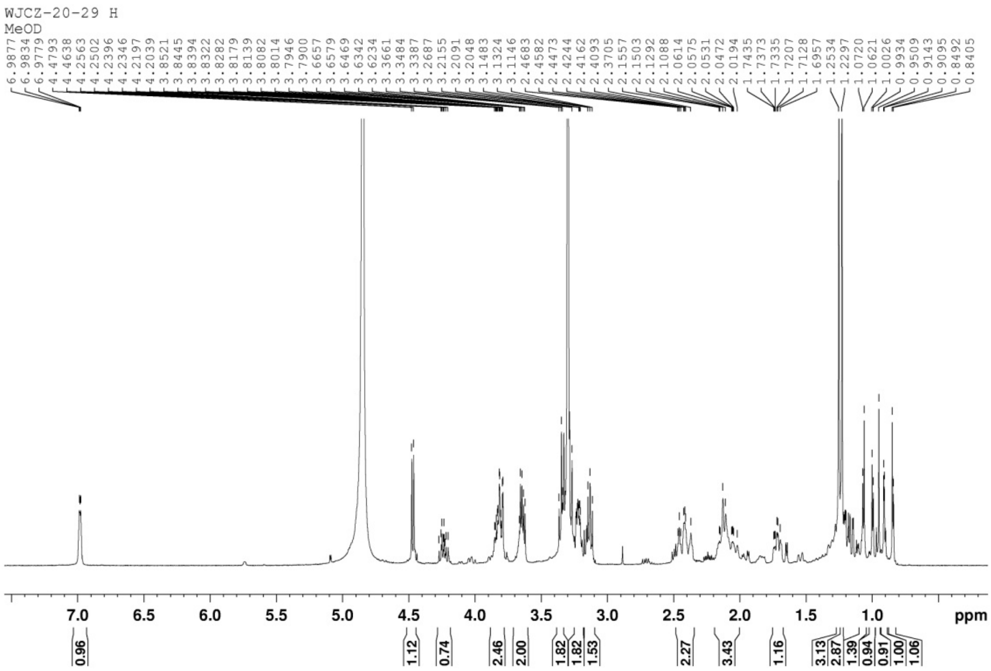

Figure S1. The ^{1}H-NMR spectrum of compound **1**.

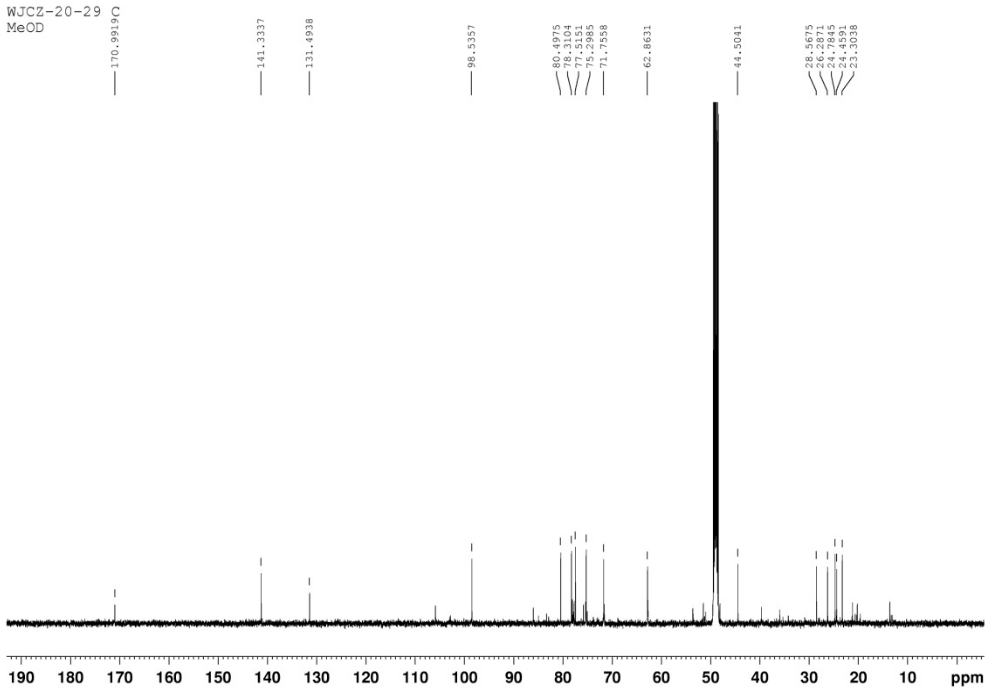

Figure S2. The ^{13}C-NMR spectrum of compound **1**.

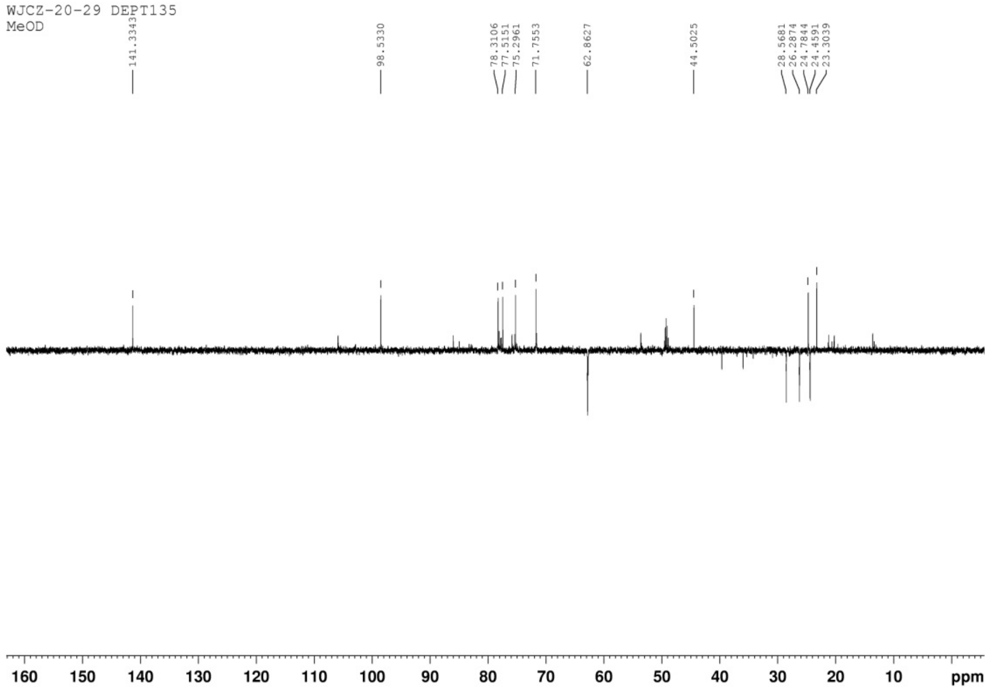

Figure S3. The DEPT 135 spectrum of compound **1**.

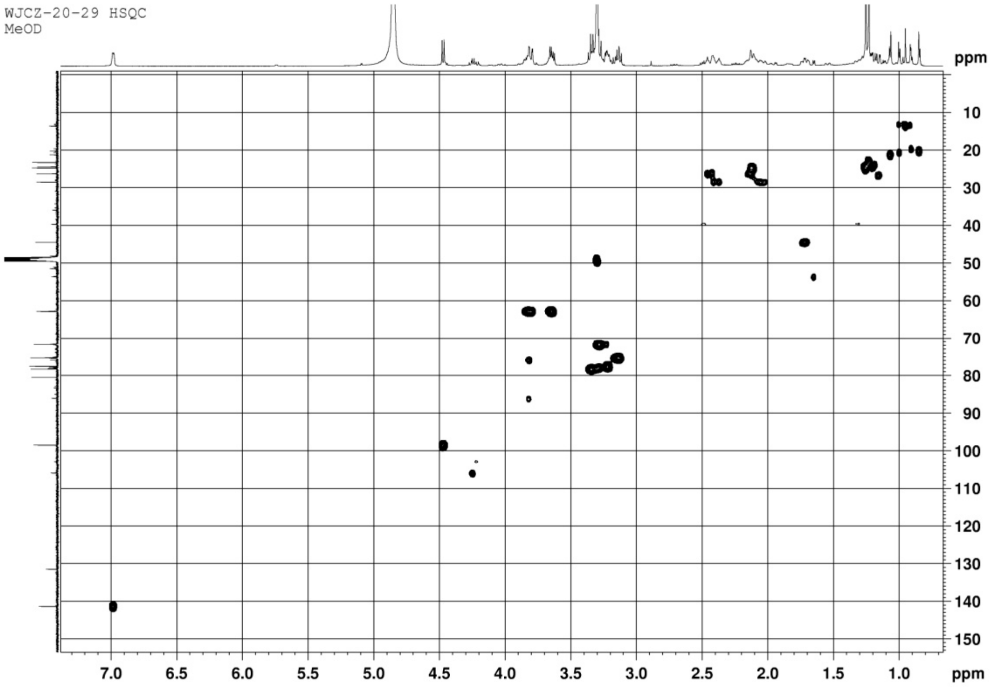

Figure S4. The HSQC spectrum of compound **1**.

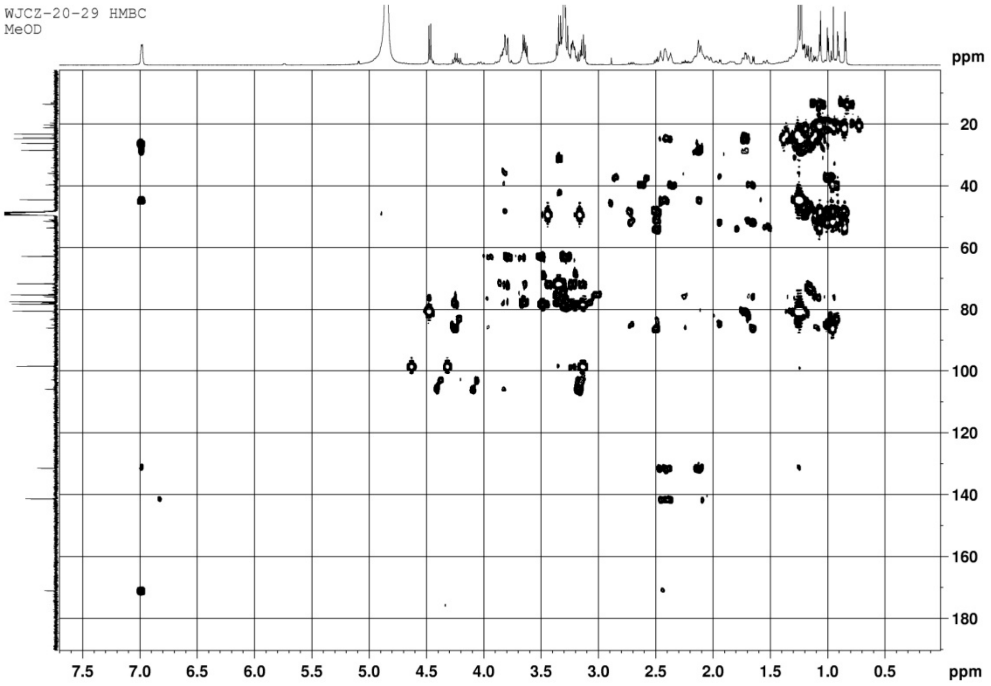

Figure S5. The HMBC spectrum of compound **1**.

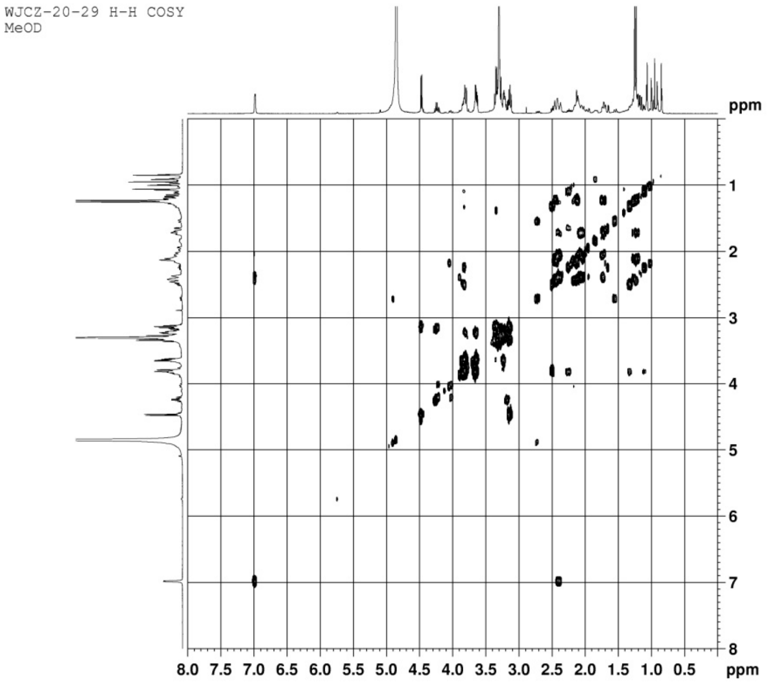

Figure S6. The ^{1}H-^{1}H COSY spectrum of compound **1**.

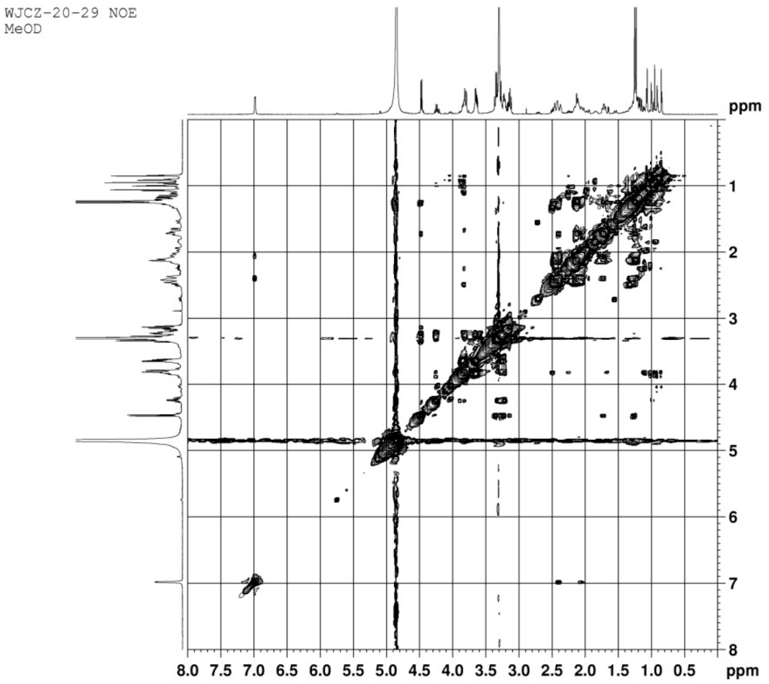

Figure S7. The NOESY spectrum of compound **1**.

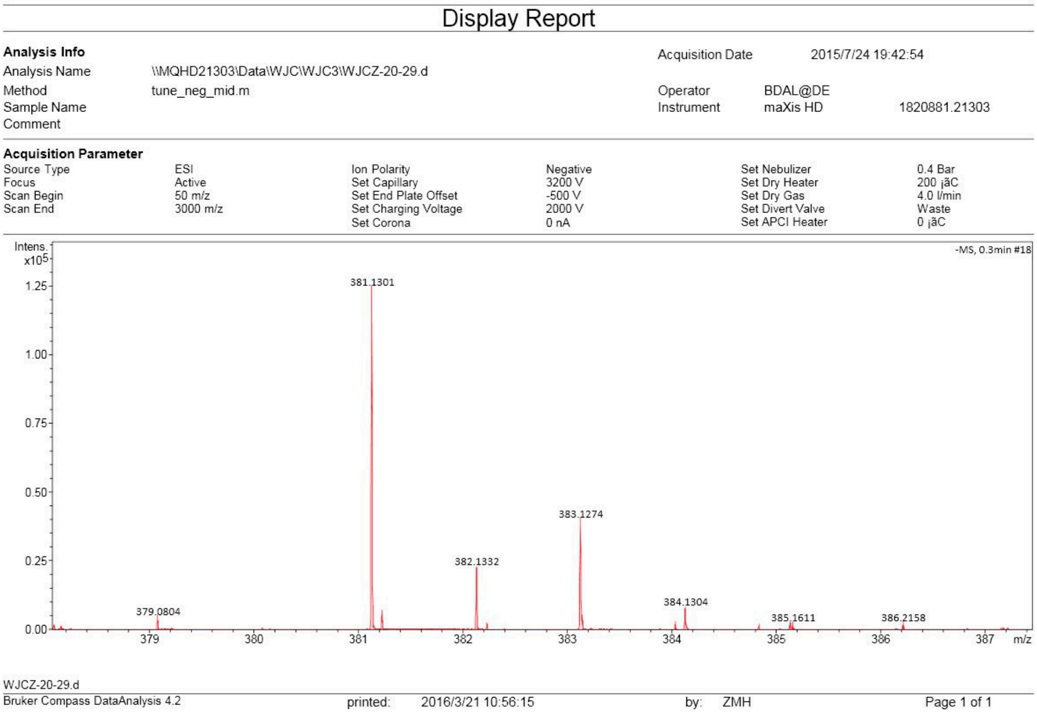

Figure S8. The HR-ESI-MS spectrum of compound **1**.

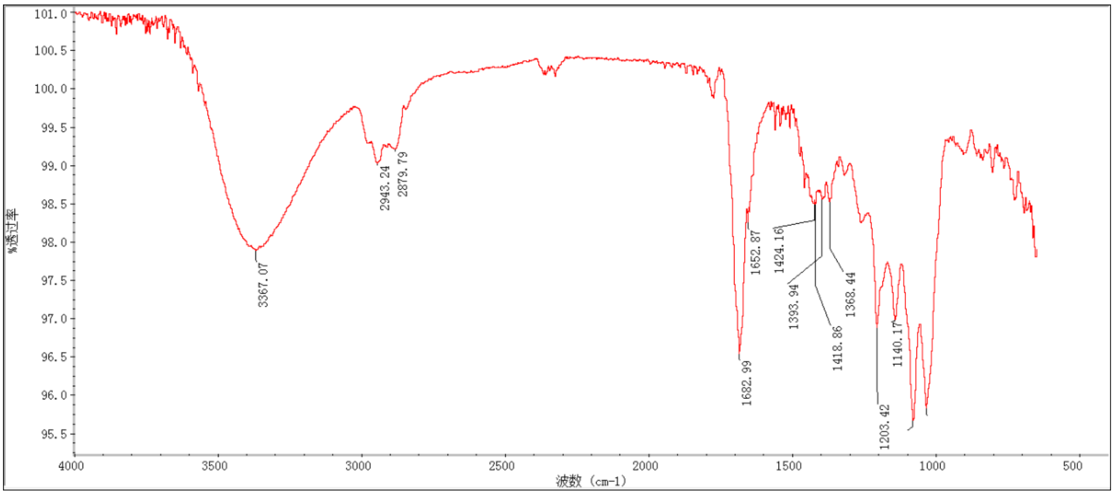

Figure S9. The IR spectrum of compound **1**.

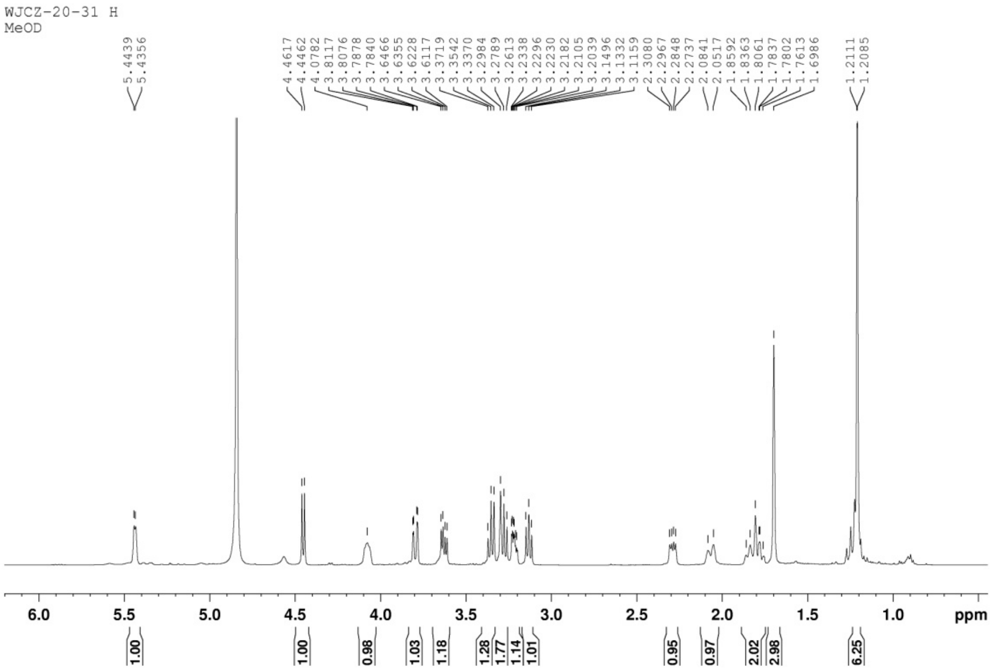

Figure S10. The ^{1}H NMR spectrum of compound **2**.

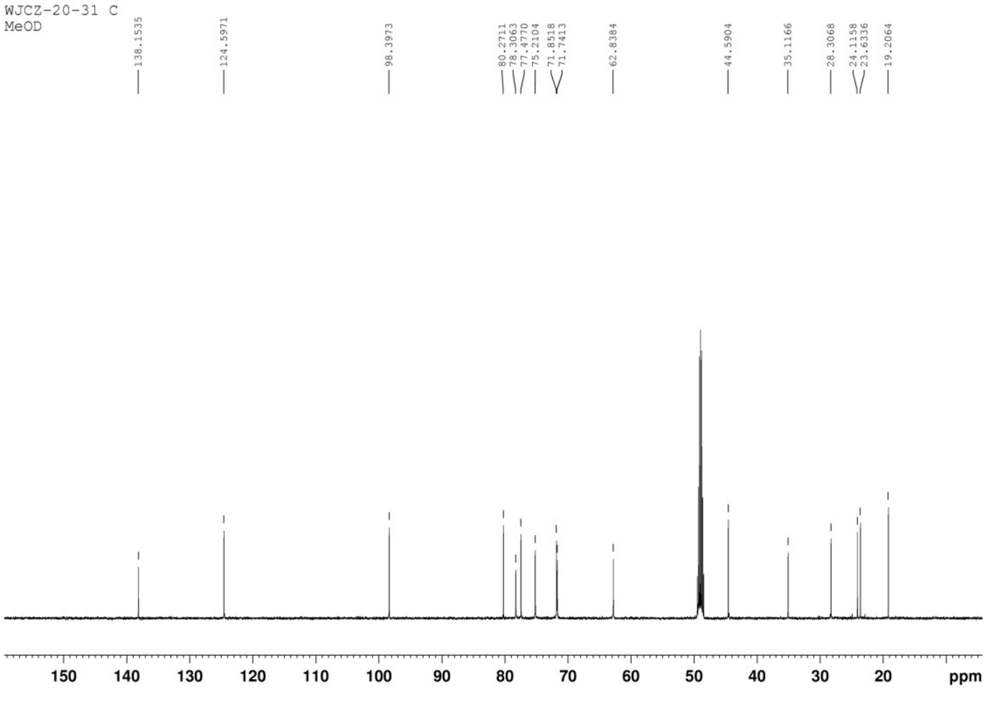

Figure S11. The ^{13}C NMR spectrum of compound **2**.

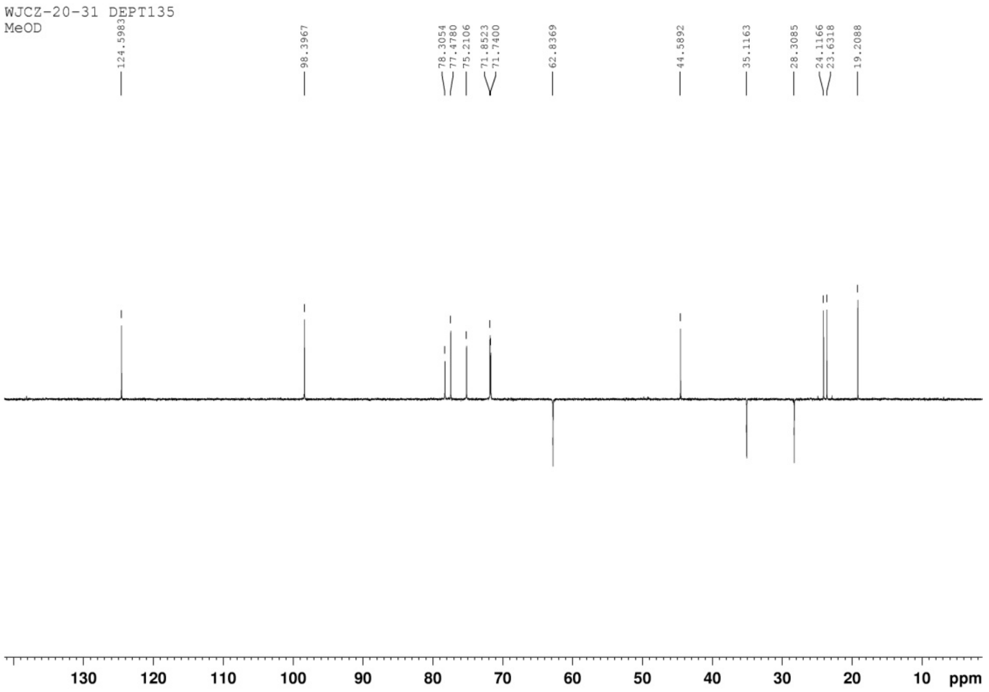

Figure S12. The DEPT 135 spectrum of compound **2**.

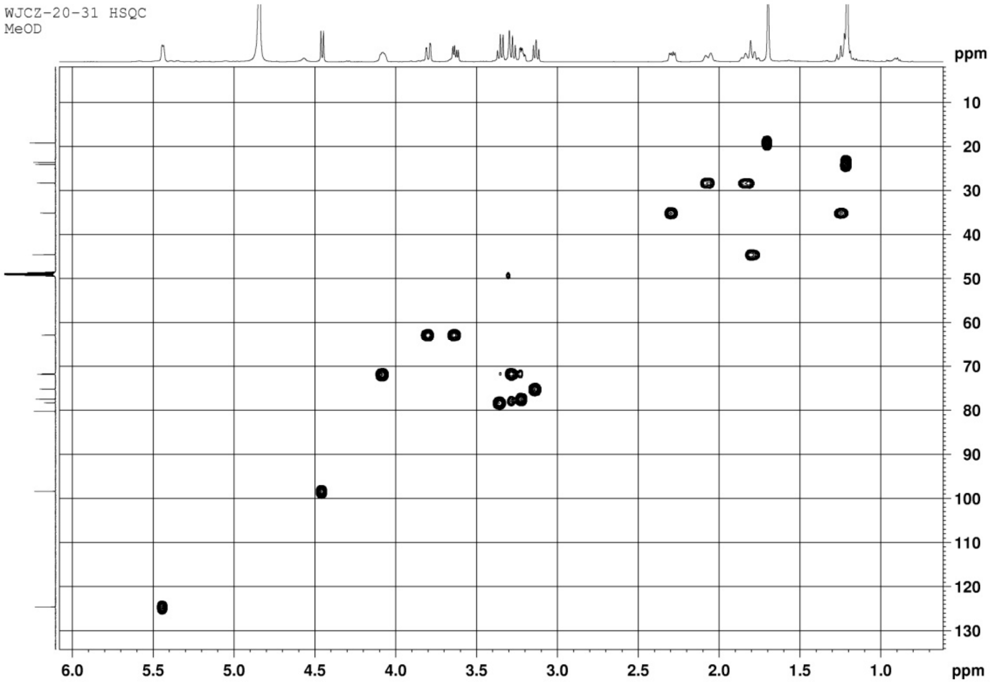

Figure S13. The HSQC spectrum of compound **2**.

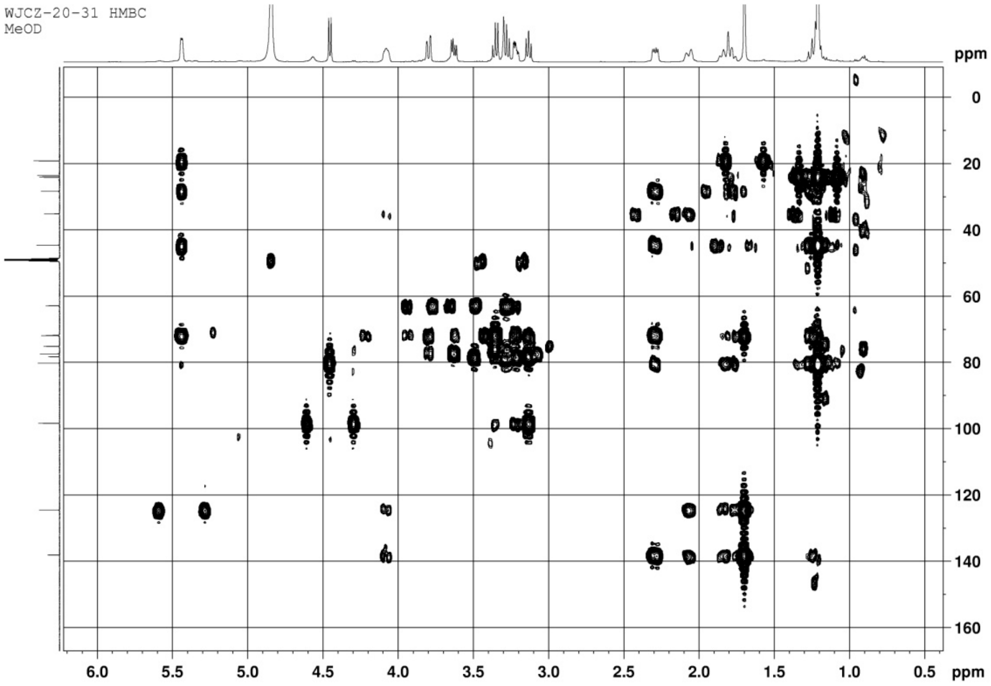

Figure S14. The HMBC spectrum of compound **2**.

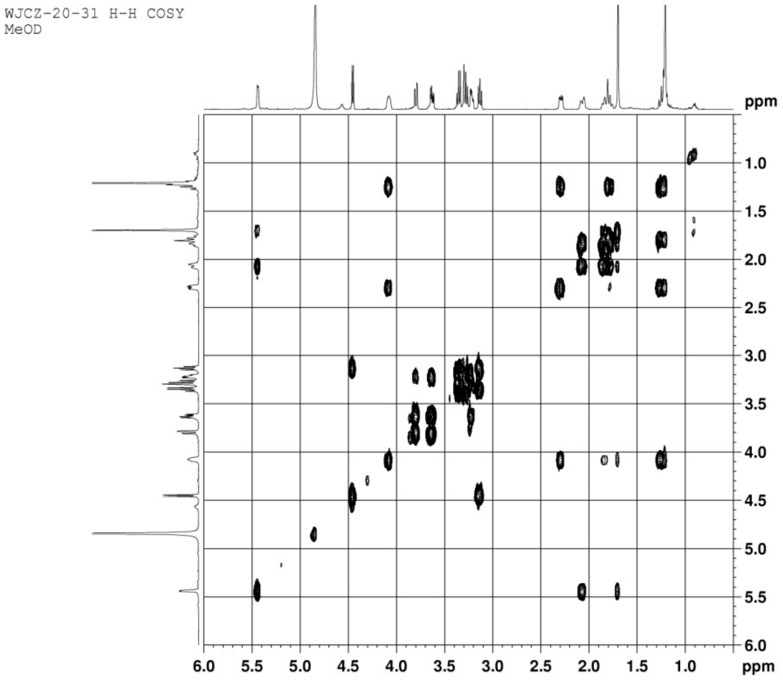

Figure S15. The ^{1}H-^{1}H COSY spectrum of compound **2**.

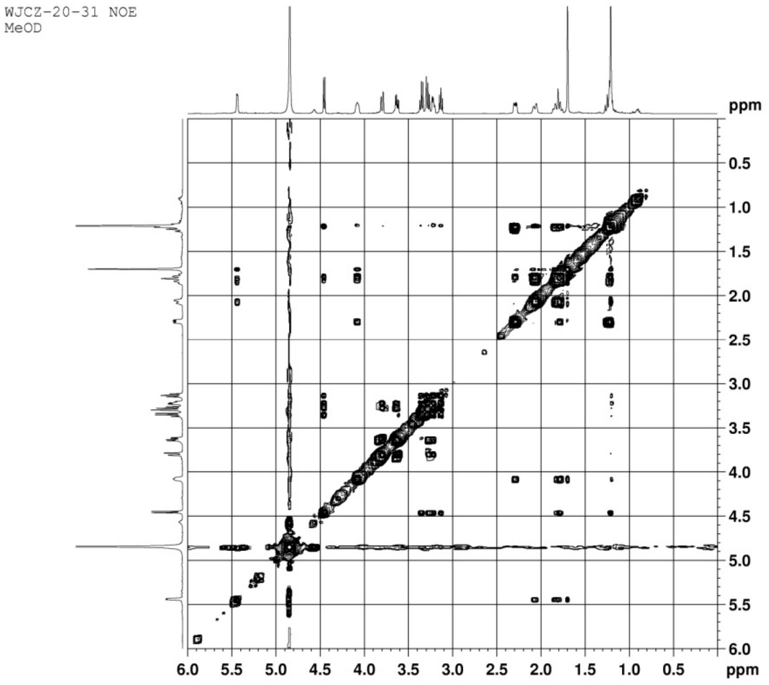

Figure S16. The NOESY spectrum of compound **2**.

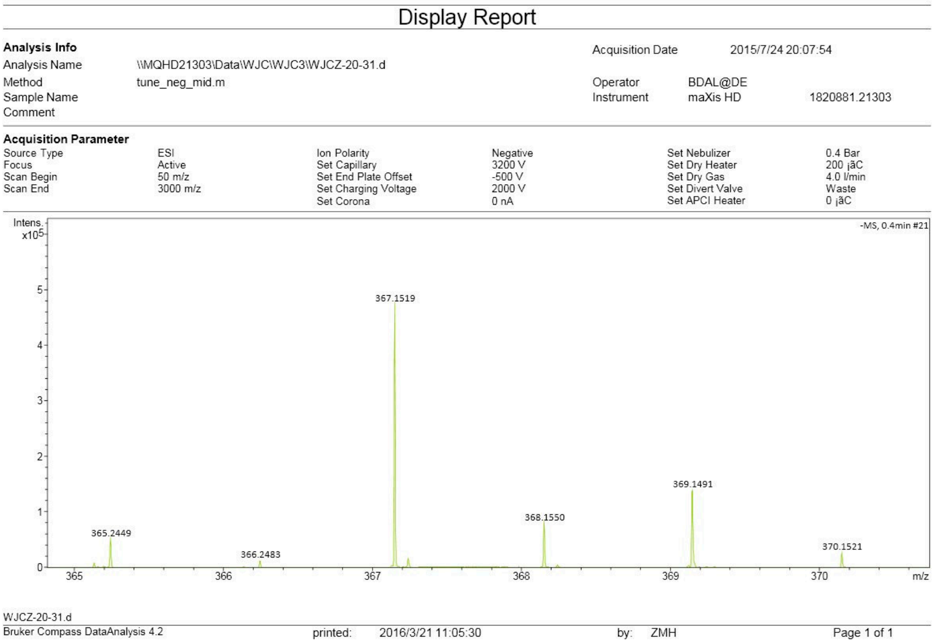

Figure S17. The HR-ESI-MS spectrum of compound **2**.

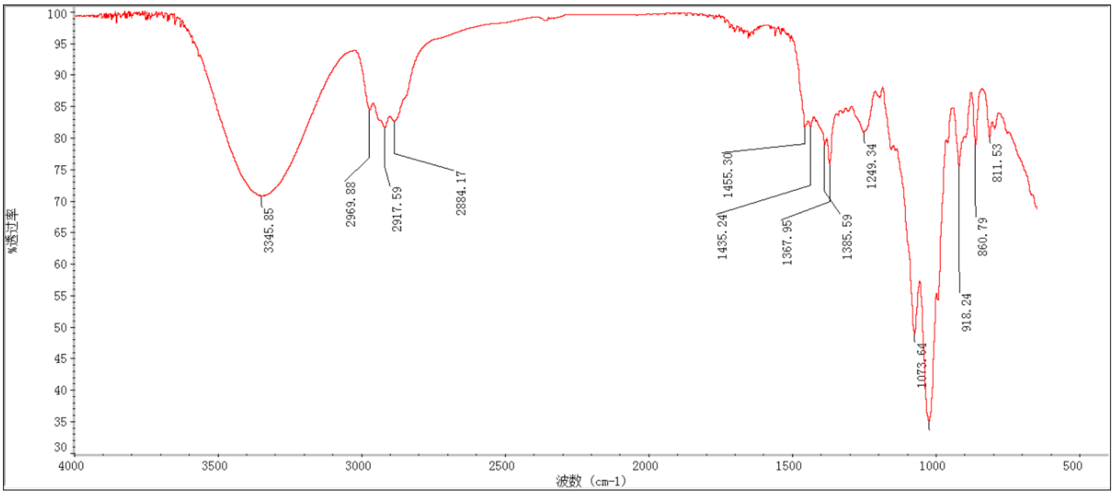

Figure S18. IR spectrum of compound **2**.

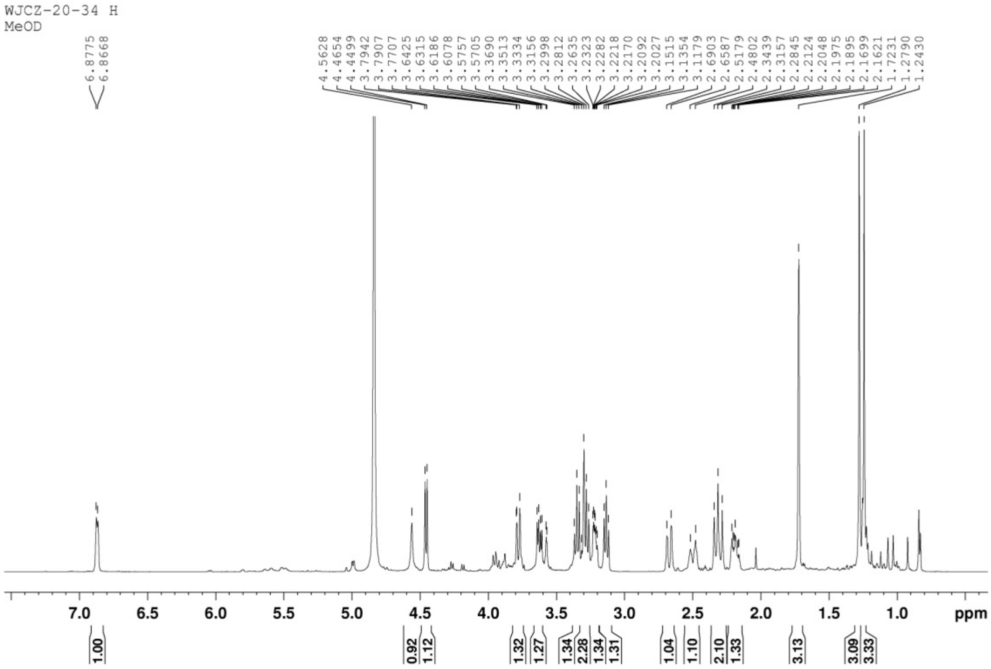

Figure S19. ^{1}H NMR spectrum of compound **3**.

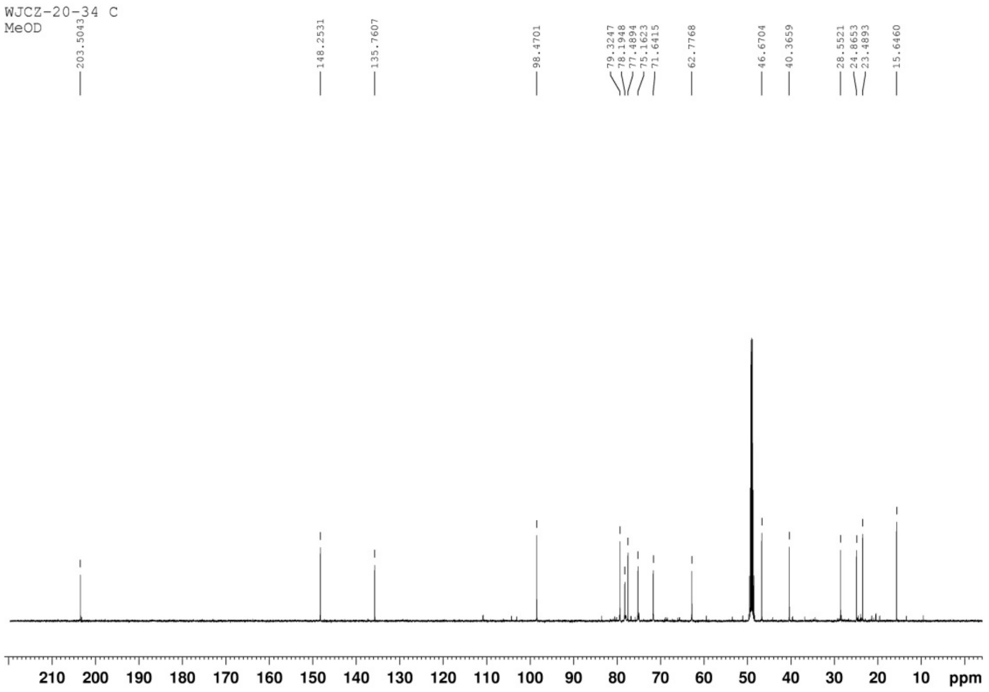

Figure S20. ^{13}C NMR spectrum of compound **3**.

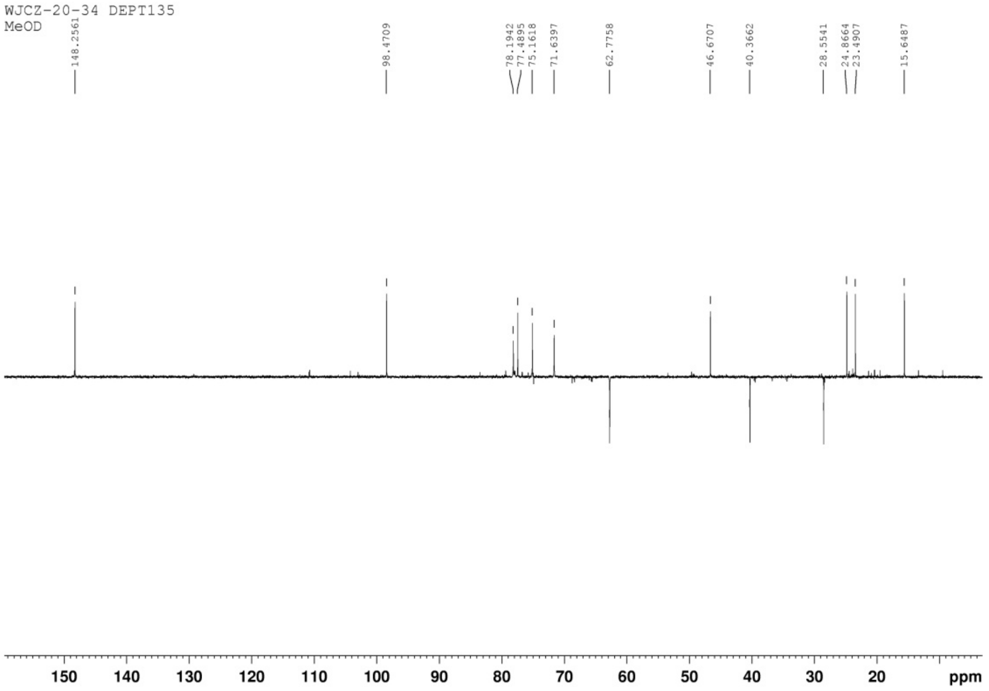

Figure S21. DEPT 135 spectrum of compound **3**.

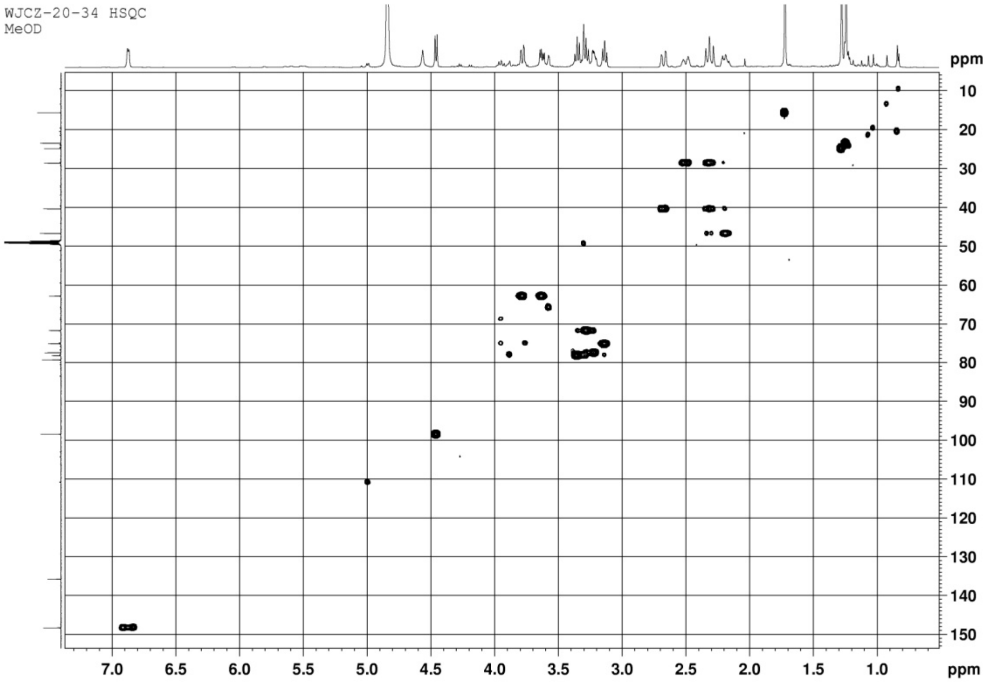

Figure S22. HSQC spectrum of compound **3**.

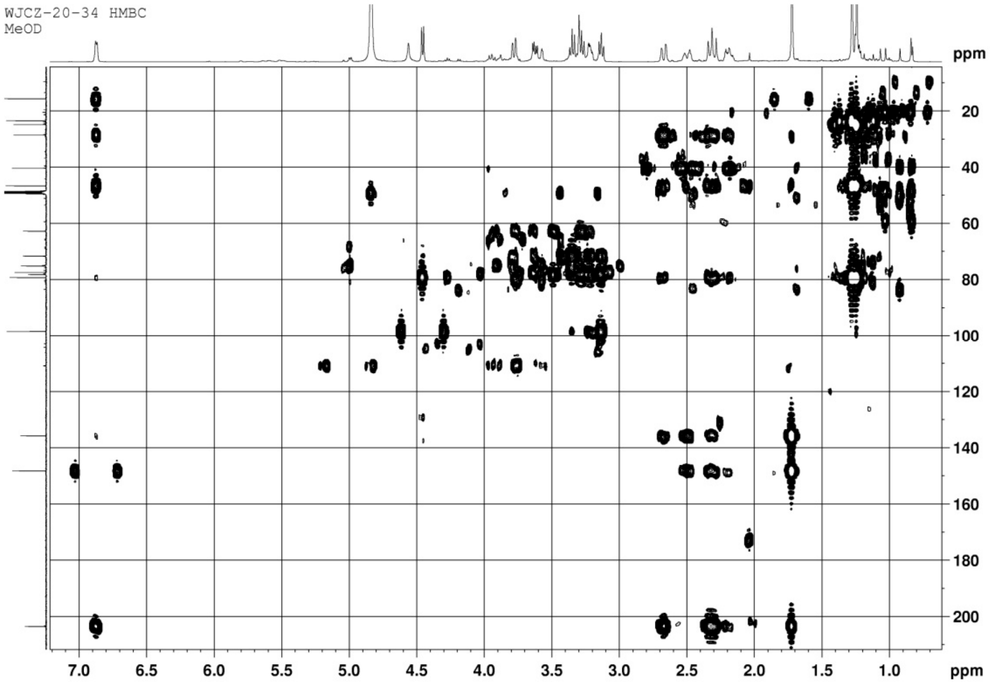

Figure S23. HMBC spectrum of compound **3**.

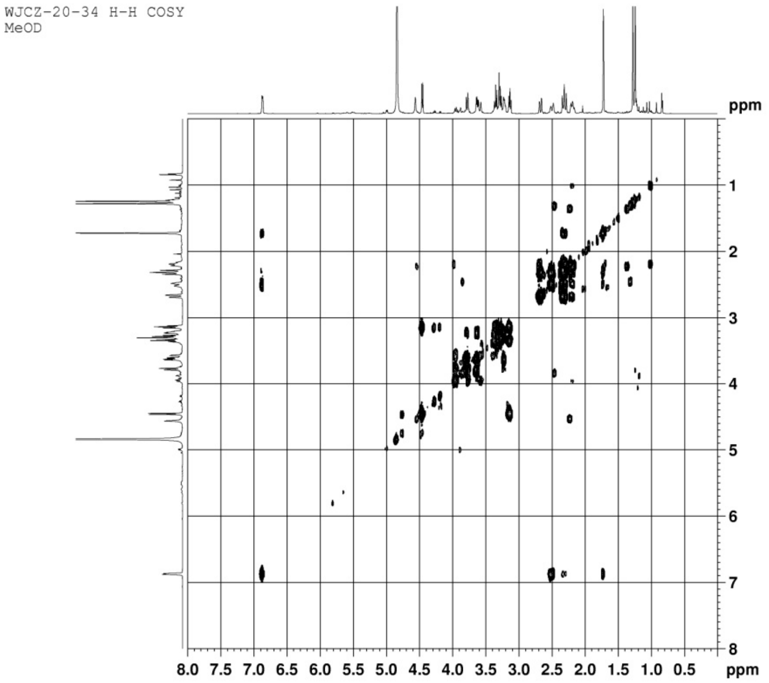

Figure S24. ^{1}H-^{1}H COSY spectrum of compound **3**.

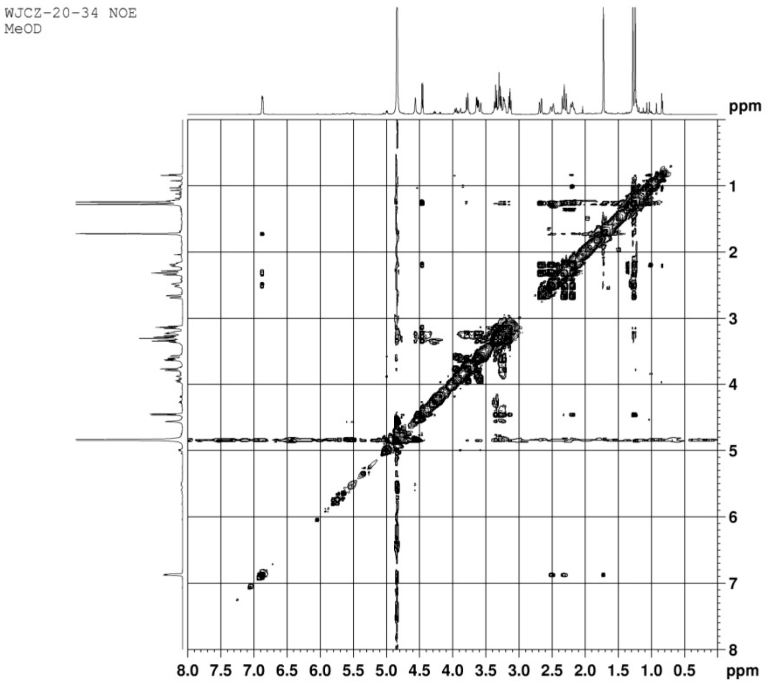

Figure S25. NOESY spectrum of compound **3**.

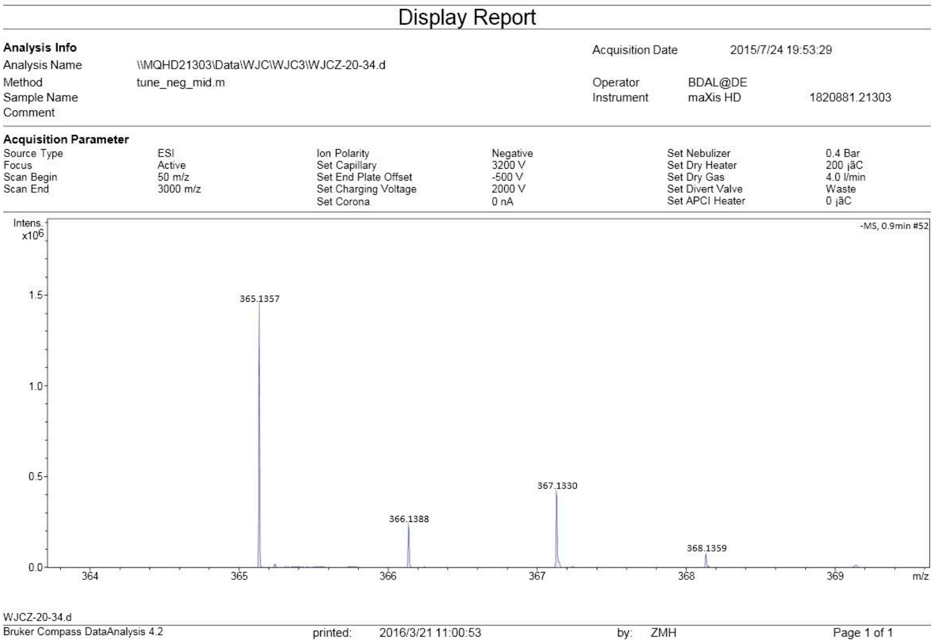

Figure S26. HR-ESI-MS spectrum of compound **3**.

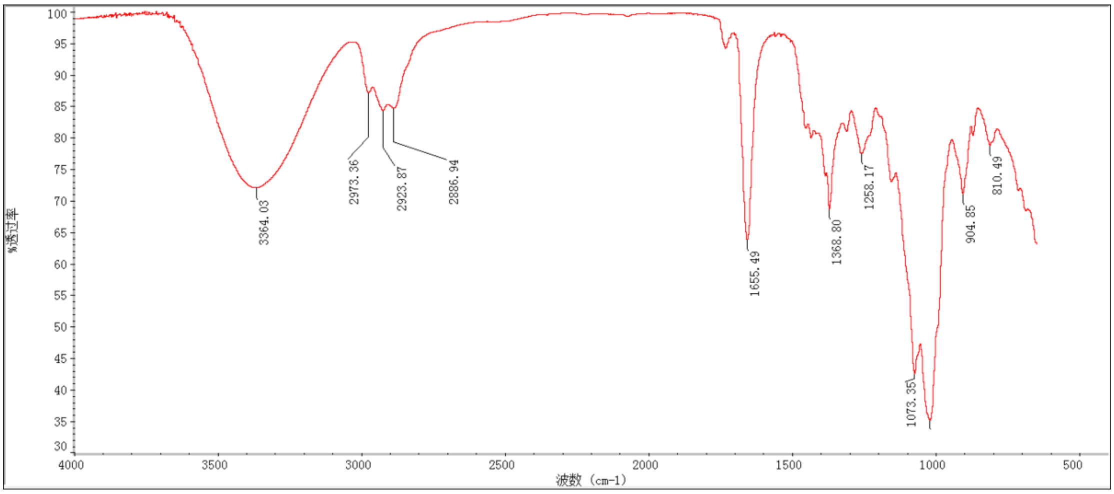

Figure S27. IR spectrum of compound **3**.

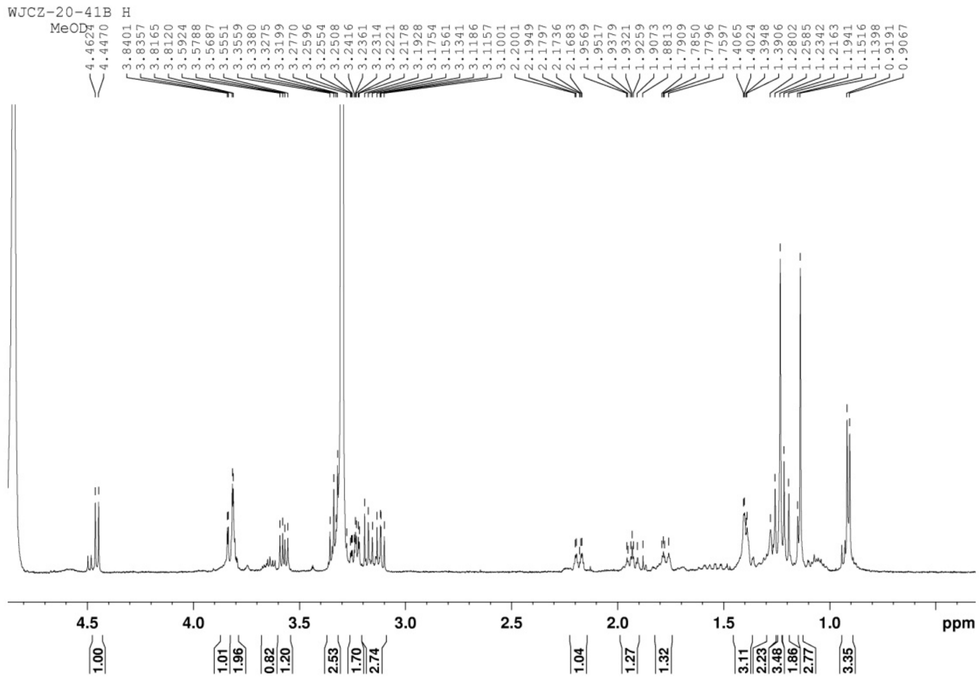

Figure S28. ^{1}H NMR spectrum of compound **4**.

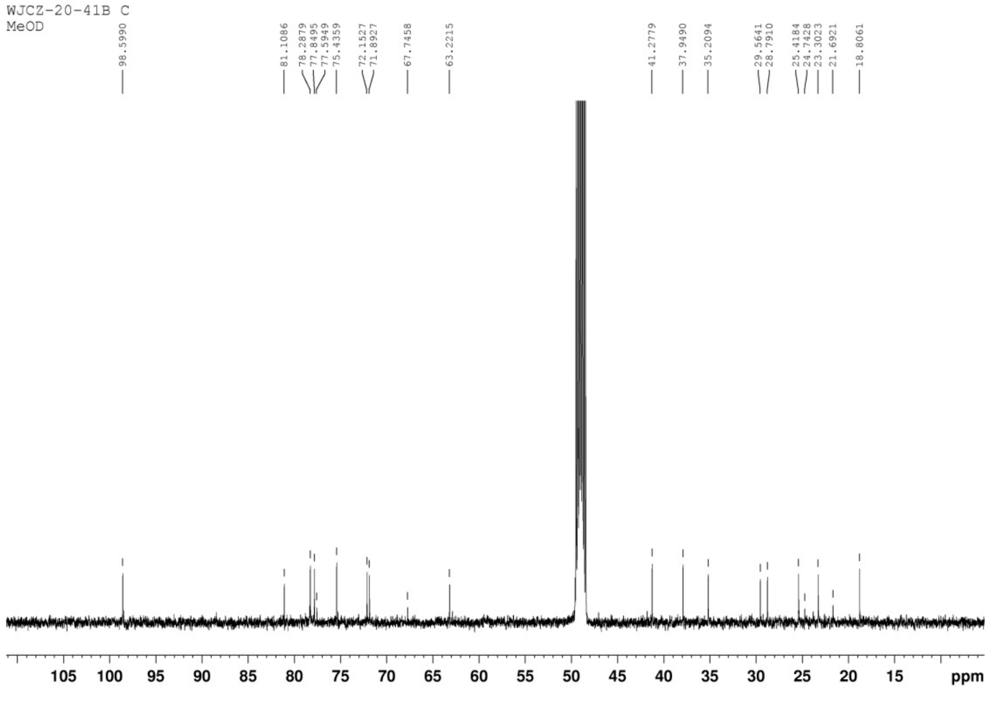

Figure S29. ^{13}C NMR spectrum of compound **4**.

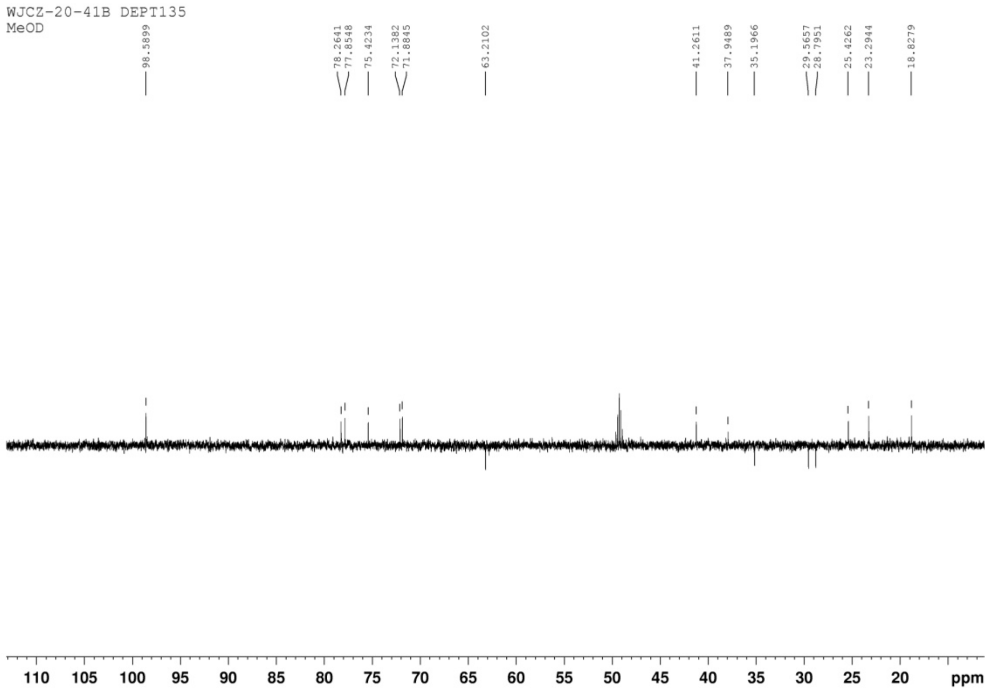

Figure S30. DEPT 135 spectrum of compound **4**.

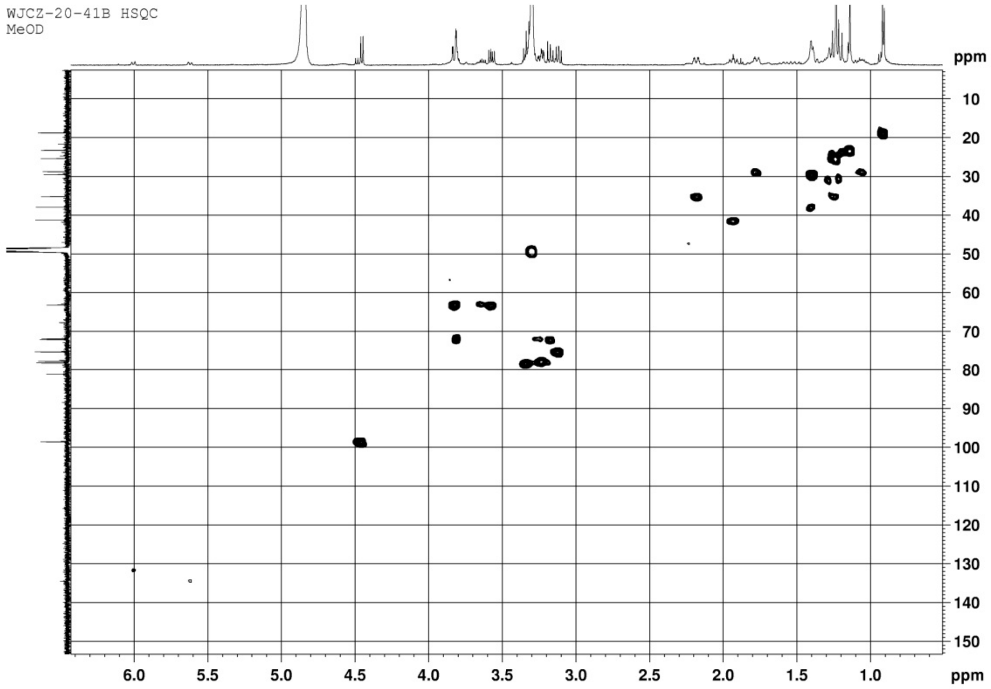

Figure S31. HSQC spectrum of compound **4**.

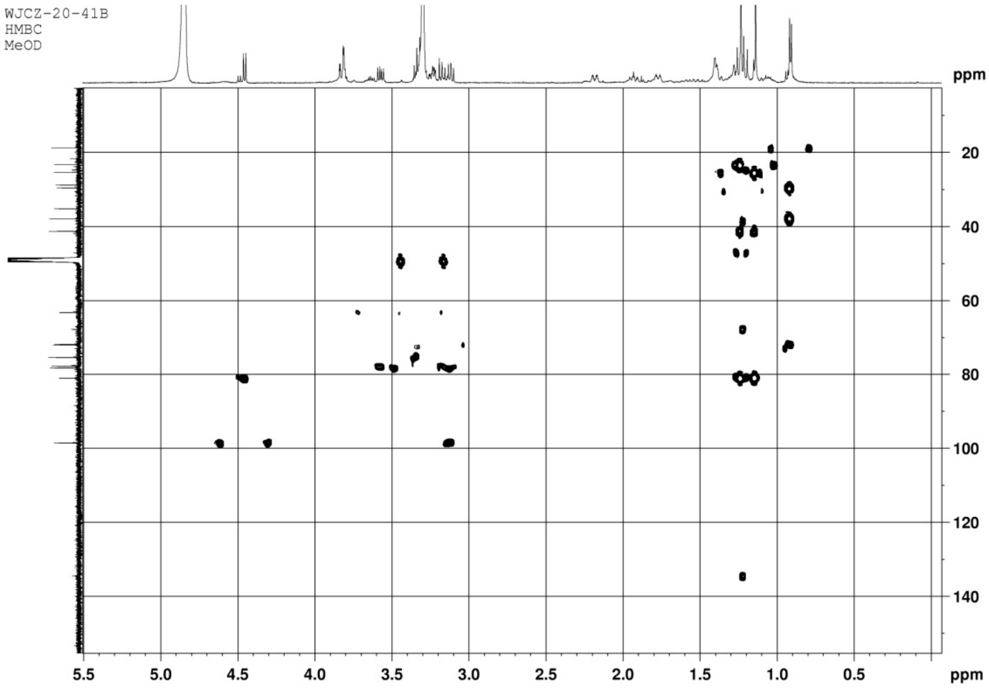

Figure S32. HMBC spectrum of compound **4**.

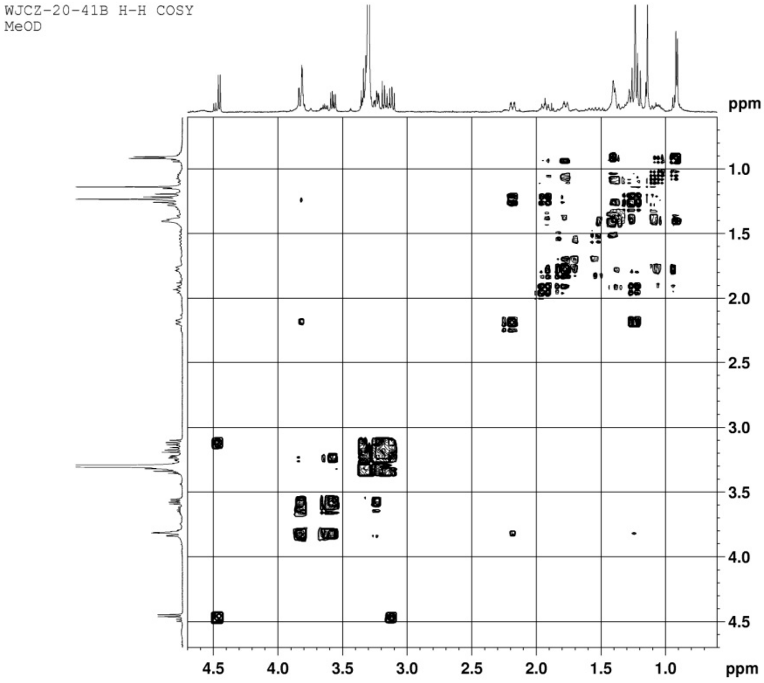

Figure S33. ^1H-^1H COSY spectrum of compound **4**.

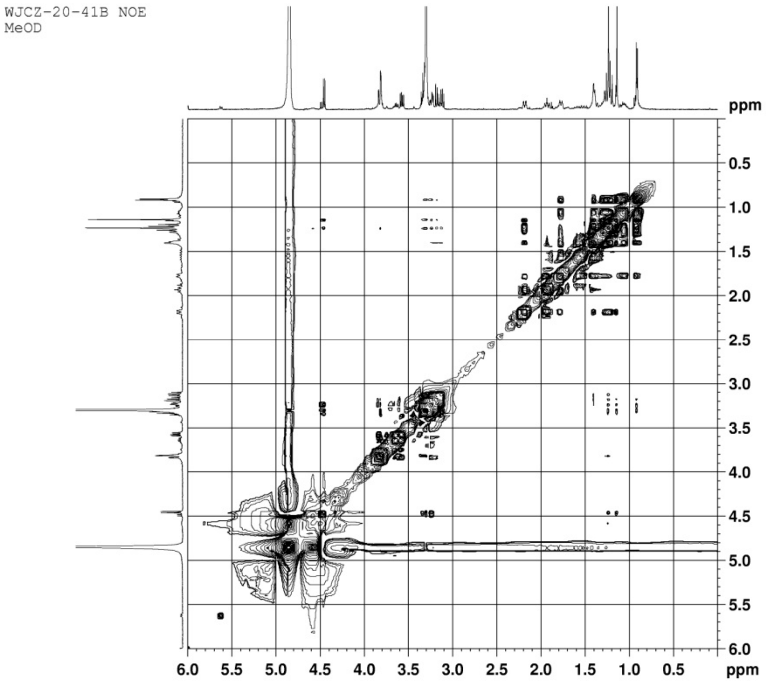

Figure S34. NOESY spectrum of compound **4**.

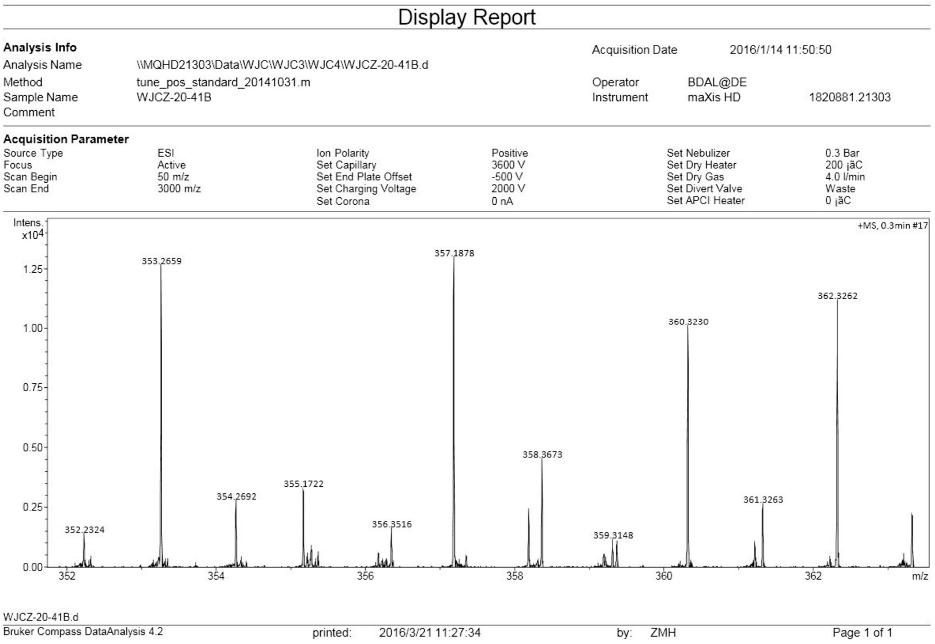

Figure S35. HR-ESI-MS spectrum of compound **4**.

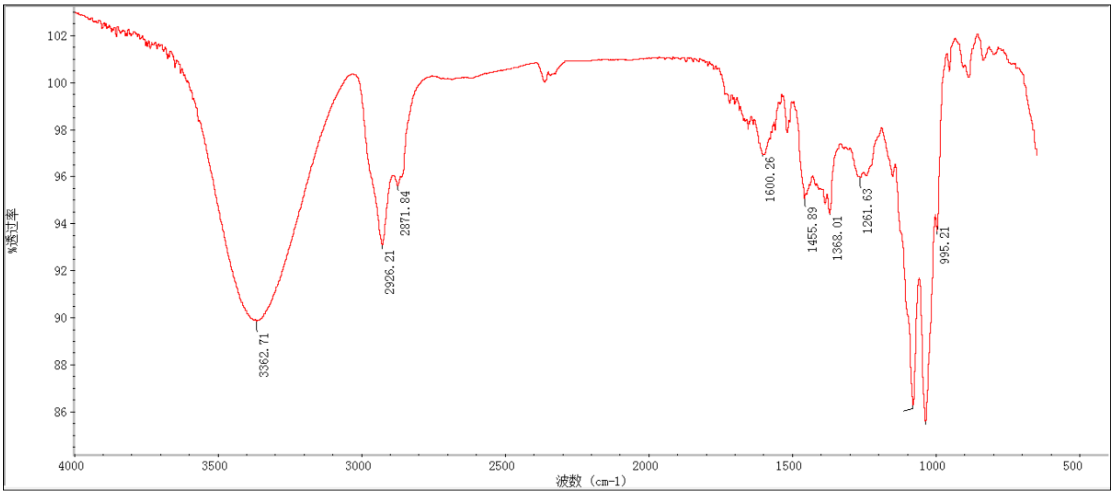

Figure S36. IR spectrum of compound **4**.
